# The psychometric performance of the EQ-HWB-9 for measuring health and wellbeing in a general population sample from Australia and New Zealand

**DOI:** 10.1007/s11136-025-04061-3

**Published:** 2025-09-18

**Authors:** Cate Bailey, Karen Trapani, Jonathan N. Davies, Nicholas Van Dam, Julieta Galante, Tessa Peasgood

**Affiliations:** 1 Melbourne Health Economics, Melbourne School of Population and Global Health, The University of Melbourne, 207 Bouverie St, Carlton, VIC 3035 Australia; 2 Contemplative Studies Centre, Melbourne School of Psychological Sciences, The University of Melbourne, Carlton, VIC Australia; 3 Centre for Health Policy, School of Population and Global Health, The University of Melbourne, Carlton, VIC Australia; 4 Sheffield Centre for Health and Related Research, School of Medicine and Population Health, University of Sheffield, Sheffield, UK

**Keywords:** EQ-HWB, Psychometrics, Validity, Caregiving, Health and wellbeing

## Abstract

**Purpose:**

The EuroQol Health and Wellbeing Short (EQ-HWB-9) is a new, generic 9-item instrument, suitable for evaluating interventions in health and social-care settings for patients and caregivers. The instrument now requires validation across general and caregiver populations. Informal caregiving can be time-intensive and impact caregiver’s physical and mental well-being. However, caregiver outcomes are often overlooked in healthcare decisions, which can lead to inefficient resource allocation. We aimed to examine the psychometric performance of the EQ-HWB-9 in a general population dataset, including caregivers of persons with disability/chronic illness.

**Methods:**

Using general population samples, stratified by age, gender, region, ancestry, and income for Australia and New Zealand, we investigated EQ-HWB-9 item distribution and known-group validity (*t*-tests; Cohen’s *d* for effect size, with sub-group analysis by country, gender and age) across sum-scores and UK pilot preference-weighted scores. Item scores were compared across caregiver groups. Convergent validity was assessed between the EQ-HWB-9 and the Kessler-6 using Spearman’s Rho.

**Results:**

The sample included 2542 participants, 2018 from Australia and 524 from New Zealand. Item distribution was similar to previous studies. Known-group validity results aligned to a priori hypotheses for caregiver, mental health, physical health and disability and sleep issues variables. Caregivers had significantly higher scores across each item than their counterparts. Convergent validity conformed to a priori expectations.

**Conclusion:**

The EQ-HWB-9 appears valid in this general population setting. This study helps to build the evidence for the use of the instrument across diverse settings. Australian- and New Zealand-specific value-sets would be a good future addition.

**Supplementary Information:**

The online version contains supplementary material available at 10.1007/s11136-025-04061-3.

## Abstract (plain language summary)

1. Why is this study needed?

The EuroQol Health and Wellbeing Short (EQ-HWB-9) is a new instrument with 9-items, intended to be an appropriate instrument for determining quality-of-life for economic evaluations in health and social-care settings. This instrument now requires validation in a range of general and caregiver populations to determine its suitability in these contexts.

2. What is the key problem/issue/question this manuscript addresses?

This study examined whether the EQ-HWB-9 was a reliable and valid instrument in a general population sample, where we could also measure differences in well-being of caregivers compared to non-caregivers. We aimed to determine whether the instrument could accurately capture differences between known groups, including caregiver, mental health, physical health and disability and sleep issues variables. We also assessed how well the instrument compared to another established mental health measure.

3. What is the main point of your study?

The study evaluated the psychometric performance of the EQ-HWB-9 in a general population sample. By analysing these datasets from Australia and New Zealand, this study tested how well the EQ-HWB-9 can distinguish between groups where we hypothesised differences would be found.

4. What are your main results and what do they mean?

The EQ-HWB-9 performed well, aligning with prior research. At the item level, the instrument effectively distinguished between caregivers and non-caregivers, with caregivers having higher scores (lower quality-of-life) on every item. All known-groups were significantly different, suggesting that the instrument is able to detect when caregiver status, mental or physical health problems and disability and sleep issues were affecting wellbeing. These findings suggest that the EQ-HWB-9 is a suitable instrument for measuring health and well-being across a range of general population and caregiver groups.

**Supplementary Information:**

The online version contains supplementary material available at 10.1007/s11136-025-04061-3.

## Introduction

The EQ Health and Wellbeing (EQ-HWB) is a new, generic instrument developed through an international collaboration, with the intention of being suitable for evaluating interventions in health, public health, and social care sectors (e.g. community and aged care) [1–3]. The instrument was specifically designed to be suitable for the measurement of caregiver impacts for inclusion in economic evaluations, where the informal costs of caregiving are often ignored [4, 5]. Informal caregiving (unpaid care provided by family members, friends, or others to individuals with health conditions, disabilities, or age-related needs) can be highly time-intensive and impact the physical and mental well-being of carers [6] (here, we distinguish unpaid care from caring for well children). When caregiver impacts are not included in economic evaluations, this can lead to sub-optimal decisions in resource-allocation [7]. These impacts are often described as “spillover effects”, and are being seen as increasingly important by decision making bodies (e.g. National Institute for Health and Care Excellence (NICE), UK [8]).

The EQ-HWB instrument has a long form with 25-items (EQ-HWB) and a short form with 9-items (EQ-HWB-9). The instrument is a valuable tool for measuring quality-of-life in a range of contexts, including for determining quality-adjusted life years (QALYs) in conjunction with a relevant value-set. A UK pilot value-set is now available for the short version, valued on the 0–1 scale suitable for calculating Quality Adjusted Life Years (QALYs) [9]. Both EQ-HWB instruments still have experimental status, and are only available for use by researchers for the purpose of validating the instruments towards releasing in a final version [10].

Recent studies presenting psychometric evidence to validate the instruments have found that the instruments appear valid in a range of caregiver populations, such as parents of children with health conditions [11], caregivers in families with young children experiencing adverse life events [12], caregivers of people living with dementia [13], informal caregivers in the United States [14], and patient and caregiver samples in China [15]. There have also been studies in the general population in Australia and China [15, 16]. These studies have assessed response distribution [11, 12, 15, 16], convergent validity [12, 14–16], known-group validity [11, 12, 14, 15], and factor structure [15, 16]. There is now also evidence on responsiveness to change [12, 17]. Whilst validation evidence is growing, there is still much work to be completed to understand how well the EQ-HWB instruments perform in a range of caregiver and other populations.

Our aims for the current study were to examine the psychometric performance of the EQ-HWB-9 in two general population datasets from Australia and New Zealand, in combination (to ascertain overall psychometrics) and separately (to investigate any differences by country). Specifically, given our overall interest in the use of the instrument in caregiver populations, we aimed to assess the distribution of the items comparing informal caregivers, (caregiving for a person with living with a disability, dementia, age-related medical conditions or elderly) to non-caregivers (non-caregivers includes those caring for well children), known-groups validity across a range of variables (including caregivers vs non-caregivers), and convergent validity of the EQ-HWB-9 items with items from a mental health distress measure, the Kessler 6 (K6) [18]. Our secondary aim was to conduct subgroup analyses analysis by country, gender and age.

## Methods

### Study design

We conducted an online cross-sectional survey of Australian and New Zealand adults. The main aim of the survey was to estimate the population prevalence of the use of meditation, yoga and other contemplative practices [19]. Data collection was based on stratified non-interlocking quota sampling for age, gender, region, ancestry, and income to represent the Australian population census from 2021 [20], and New Zealand for 2018 [21]. Ethics approval was from the University of Melbourne Human Research Ethics Committee (#23,004). Participants were recruited and consented through the Qualtrics online platform. Data acquisition was managed by Qualtrics, with guidance from the authors. Data collection was collected between September 2023 and January 2024.

### Participants

Participants were adults (aged 18 +) from the general population of Australia and New Zealand. Participants were excluded if they did not disclose information relative to the stratification, or if they were deemed fraudulent (See our paper presenting baseline results for further information [19]). Participants were reimbursed for their time.

### Materials

The study dataset included caregiver status, mental health (K6), chronic health, disability status, sleep problems, and household income. The EQ-HWB-9 instrument was used with permission and scored two different ways: we applied preference-weights from the pilot UK value-set to the EQ-HWB-9 scores to produce EQ-HWB-9 index-scores [9], and we summed the EQ-HWB-9 responses to produce sum-scores (i.e. total scores: EQ-HWB-9 items were scored on a 1–5 scale).

The Caregiver variable question was derived from the following multiple-choice question: Do you provide unpaid care for any of the following? (select all that apply): 1) My own child(ren), 2) Child(ren) other than my own, 3) Elderly family member(s), 4) Family member(s) with a disability and/or long-term illness, 5) Family member(s) with dementia or problems related to old age, 6) Non-family member(s) with a disability and/or long-term illness, 7) Non-family member(s) with dementia or problems related to old age, and 8) None of the above (I don’t provide unpaid care for anyone). We created an informal caregiver variable (yes/no) with informal caregivers defined as anyone providing unpaid care for elderly family members, family members and non-family members with a disability and/or long-term illness and/or dementia and/or problems related to old age (i.e., those selecting options 3, 4, 5, 6 and/or 7), compared to non-caregivers (i.e., those providing unpaid care for well children or not providing unpaid care for anyone, options 1 and/or 2; or 8). Hence, our caregiver variable represents people caring for those who are elderly, and/or with a chronic illness or disability, but does not include parents of healthy children (unless they also select options 4–7).

Mental health was measured using the K6 [22], which was developed as a screening tool for serious mental illness and mental distress. The K6 has 6 items: felt nervous, hopeless, restless or fidgety, depressed, everything was an effort, and felt worthless. We used the standard two-group cut-points: ‘probable’ (scores of 6–18) versus ‘no probable’ (scores of 19–30) mental distress [18].

Chronic health condition was defined using the question: “Do you have any of the following chronic physical health conditions? (select all that apply)” with the options for arthritis, asthma, chronic pain (i.e., pain that is persistent or recurrent for longer than 3 months; including musculoskeletal pain, visceral pain, headache), cancer, cardiovascular disease, chronic obstructive pulmonary disease, diabetes, other chronic physical health condition, none of these. The question was recoded to “any of the first seven responses”, versus “no health condition”.

Disability was defined using the question: “Do you identify as having a disability?” (yes/no). Sleep issues were defined using the question: “In the last 7 days, did you have problems with your sleep?” with the response options of: none of the time, only occasionally, sometimes, often, and most or all of the time and recoded to none of the time/only occasionally vs sometimes/often/most or all of the time. The sleep item was item 6 of the full 25-item EQ-HWB instrument [23], used with permission. Low income was derived from the following question: “What was your total household income before taxes during the past 12 months?” Income was defined in bands as per the county’s census data [20, 21]. Low income in Australia was defined as AU$52,000 or less, and in New Zealand as NZ$50,000 or less. Gender was defined as two groups (female/male) by removing “non-binary/other” which was 0.4% of the sample. Age groups were coded from continuous data for the groups: 18–29, 30–39, 40–49, 50–59, and 60 + years. LGBTQIA + was defined using the question: “Do you identify as being LGBTQIA + ? (i.e., lesbian, gay, bisexual, transgender, intersex, queer, asexual or other sexually or gender diverse)”.

### Sample size

We aimed to recruit 2000 participants from Australia and 500 from New Zealand. There is no defined sample size for psychometric analysis, however Frost et al. suggest that 200 cases may be suitable for testing reliability and validity [24]. We anticipated that for both countries, our sample size would significantly exceed the number of participants required for psychometric analysis, allowing us to conduct known-groups analysis by subgroup. Differences in sample size between countries was due to the primary aim of the main study and also reflected the countries' different population sizes.

### Statistical analysis

The psychometric properties of the EQ-HWB-9 were assessed in terms of distribution, known groups validity analysis with subgroup analysis by gender and age, and convergent analysis against the K6. We used number and percentage per response per question for the distribution, and included means and standard deviations, noting that the data is ordinal. The base analysis combined the data from both countries to determine psychometric outcomes across the full sample, and we conducted further analysis by country to determine whether the results differed.

For the known groups validity analyses, we used *t*-tests with Cohen’s *d* as the effect size (0.2–0.49 = small, 0.5–0.79 = moderate, above 0.8 = large [25]). We hypothesised that caregivers would have lower quality-of-life (higher sum-scores, lower index-scores) than non-caregivers [26]. We hypothesised that participants with higher mental distress [12], chronic health conditions [11] or, disability [27], sleep issues [28], or low income [29] would have lower quality-of-life than their counterparts. We conducted subgroup analyses by gender and age.

To investigate convergent validity, we compared item scores from the EQ-HWB-9 to the K6 using Spearman correlations for ordinal data. We included EQ-HWB-9 and K6 items, plus EQ-HWB-9, sum- and index-scores and K6 total scores. We defined correlation strength as 0.1–0.29 considered weak, 0.3–0.49 moderate, and > 0.5 strong [25]. We made a priori hypotheses regarding the correlations that we expected to be moderate (0.3) or above, as per our previous study [12].

## Results

### Baseline characteristics

The final sample included 2542 participants, with 2018 from Australia and 524 from New Zealand (NZ). There were no missing data due to participants being required to complete the entire survey. Caregivers, as defined, made up 18.8% of the sample. Baseline characteristics for informal caregivers (caring for those with a disability, long-term illness, dementia and/or problems related to old age, and/or elderly family member), non-caregivers and totals are presented in Table 1, with tests of statistical significance. Caregivers were significantly more likely to work full-time and less likely to be retired, more likely to identify as LGBTQIA+ , have mental health distress (higher K6 total scores), a chronic health condition, sleep problems, and lower age than non-caregivers. There were no differences between these groups on gender, household composition, disability status, or indigenous status. Baseline characteristics by country can be found in Tables S1a and S1b in the supplementary files. There were some dissimilarities in outcomes in the NZ dataset, where there were no significant differences between caregivers and non-caregivers on employment status, mental distress, LGBTQIA+ , chronic health condition or sleep problems. The only observed difference between groups in NZ was for income, with caregivers more likely to have low income.

**Table 1:** Baseline characteristics for combined Australia and New Zealand by caregiver status and totals, with significance tests between groups

	Caregivers*	Non-caregivers	Total	χ2 (df)	*p*-value
	(n = 479)	(n = 2063)	(n = 2542)		
	# (%)	# (%)	# (%)		
Sex				**1.12 (2)**	**0.57**
Female	240 (50.10)	1072 (51.96)	1312 (51.61)		
Male	238 (49.69)	982 (47.60)	1220 (47.99)		
Other (All)	1 (0.21)	9 (0.44)	10 (0.39)		
Education				**24.75 (7)**	**0.001**
Less than Primary	1 (0.21)	3 (0.15)	4 (0.16)		
Primary	2 (0.42)	10 (0.48)	12 (0.47)		
Some Secondary	23 (4.80)	180 (8.73)	203 (7.99)		
Secondary	69 (14.41)	387 (18.76)	456 (17.94)		
Vocational or Similar	100 (20.88)	440 (21.33)	540 (21.24)		
Some University but no degree	83 (17.33)	230 (11.15)	313 (12.31)		
University—Bachelors Degree	150 (31.32)	621 (30.10)	771 (30.33)		
Graduate or professional degree	51 (10.65)	192 (9.31)	243 (9.56)		
Employment				**56.03 (6)**	**< 0.001**
Working full-time	312 (65.14)	966 (46.83)	1278 (50.28)		
Working part-time	66 (13.78)	369 (17.89)	435 (17.11)		
Studying	8 (1.67)	62 (3.01)	70 (2.75)		
Unemployed and looking for work	15 (3.13)	99 (4.80)	114 (4.48)		
Unemployed and not looking for work	13 (2.71)	83 (4.02)	96 (3.78)		
Retired	53 (11.06)	430 (20.84)	483 (19.00)		
Other	12 (2.51)	54 (2.62)	66 (2.60)		
Household composition				**1.30 (4)**	**0.862**
Alone	105 (21.92)	436 (21.13)	541 (21.28)		
Single parent	53 (11.06)	216 (10.47)	269 (10.58)		
Couple with children	112 (23.38)	456 (22.10)	568 (22.34)		
Couple	144 (30.06)	672 (32.57)	816 (32.10)		
Other	65 (13.57)	283 (13.72)	348 (13.69)		
Identifies as LGBTQIA+				**152.06 (2)**	**< 0.001**
No	325 (67.85)	1842 (89.29)	2167 (85.25)		
Yes	152 (31.73)	206 (9.99)	358 (14.08)		
Prefer not to say	2 (0.42)	15 (0.73)	17 (0.67)		
Mental distress				**31.72 (1)**	**< 0.001**
No	336 (70.15)	1685 (81.68)	2021 (79.50)		
Yes	143 (29.85)	378 (18.32)	521 (20.50)		
Chronic health condition				**73.15 (1)**	**< 0.001**
No	162 (33.82)	1145 (55.50)	1307 (51.42)		
Yes	317 (66.18)	918 (44.50)	1235 (48.58)		
Disability				**0.00 (1)**	**0.985**
No or refer not to say	416 (86.85)	1791 (86.82)	2207 (86.82)		
Yes	63 (13.15)	272 (13.18)	335 (13.18)		
Sleep issues				**36.80 (1)**	**< 0.001**
None of the time or Only occasionally	197 (41.13)	1165 (56.47)	1362 (53.58)		
Sometimes, Often, Most or all of the time	282 (58.87)	898 (43.53)	1180 (46.42)		
Income				**9.97 (1)**	**0.002**
Other Income	380 (79.33)	1491 (72.27)	1871 (73.60)		
Low Income	99 (20.67)	572 (27.73)	671 (26.40)		
Indigenous status (AU)				**0.32 (1)**	**0.573**
Non-Indigenous	408 (96.45)	1547 (96.99)	1955 (96.88)		
Indigenous	15 (3.55)	48 (3.01)	63 (3.12)		
Indigenous status (NZ)				**6.14 (4)**	**0.189**
Non-Indigenous	39 (69.64)	358 (76.50)	397 (75.76)		
Indigenous	17 (29.36)	110 (23.51)	127 (24.24)		

K6 total score	Mean (SD)	Mean (SD)	Mean (SD)	*t* (df)	*p*-value
	14.97 (5.34)	12.91 (5.86)	13.3 (5.82)	**− 7.04 (2540)**	**< 0.001**
Age (continuous)	44.08 (15.56)	47.35 (18.22)	46.73 (17.79)	**3.62 (2540)**	**< 0.001**

*Caregiving for persons with a disability and/or long-term illness, and/or dementia and/or problems related to old age, and/or elderly family member, compared to all other participants

### Distribution

**Fig. 1:**
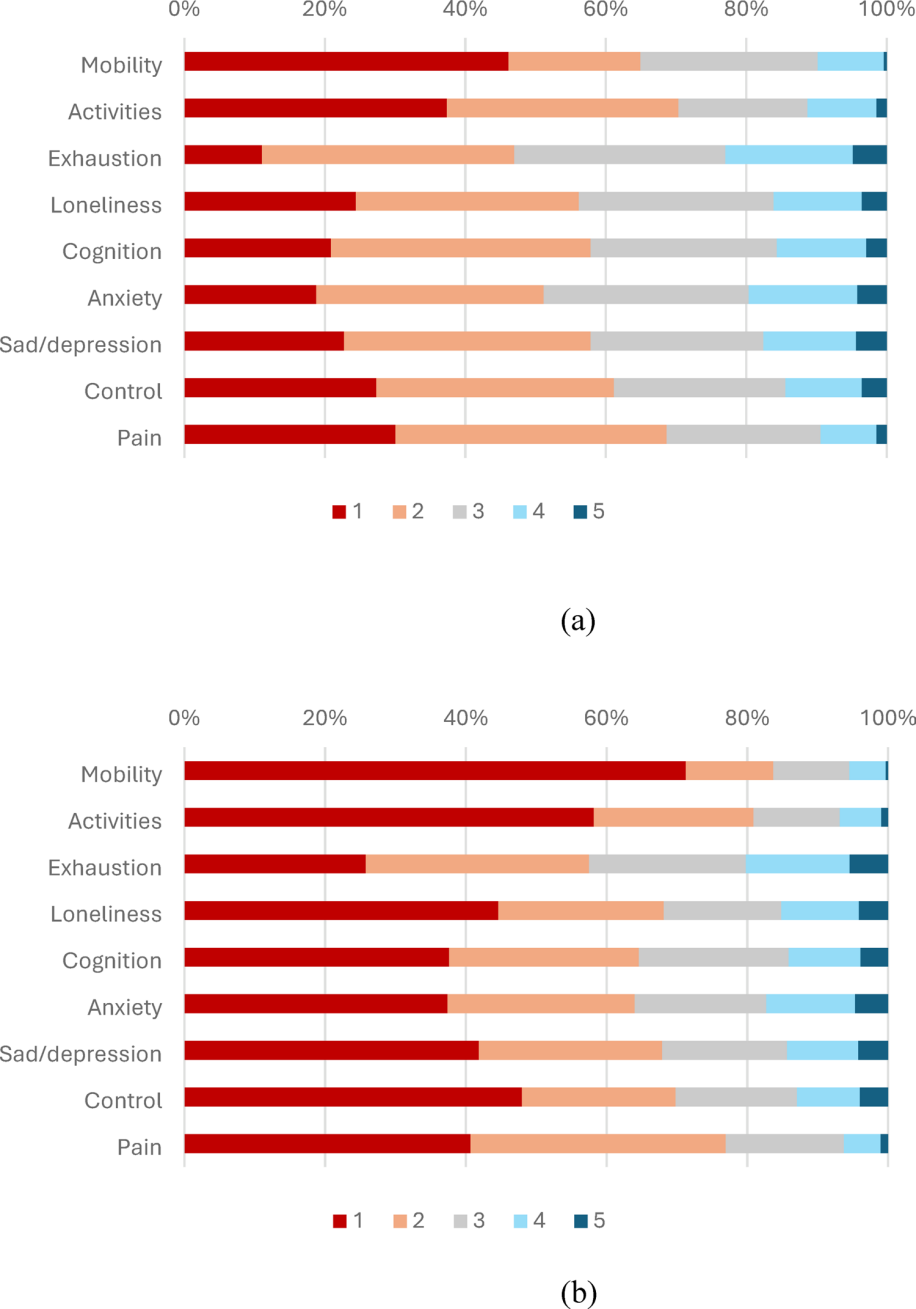
**a** Distribution of EQ-HWB-9 item responses for caregivers  2.. **b** Distribution of EQ-HWB-9 item responses for non-caregivers (legend numbers match the responses displayed in Table 2).

Response distribution scores (number and percentage) for the EQ-HWB-9 items are presented in Table 2 by caregiver group and full sample, with percentage difference scores between caregiver groups, means, and standard deviations (SD) (noting the ordinal data). Data distribution for the caregiver/non-caregiver samples is displayed pictorially in Fig. 1a and b. The percentage difference scores between groups demonstrate that the caregivers had lower scores on all 9 items. Responses on the mobility and activities items indicated that there were lower problems in these domains. Distribution by country is shown in Tables S2a and S2b. There were larger differences between caregiver and non-caregiver groups for the Australian data for all items than the New Zealand data. A density plot of the EQ-HWB-9 index scores is shown in Fig. S1.

**Table 2:** Distribution of EQ-HWB-9 item responses (number and percentage) by caregivers, non-caregivers and full sample, with percentage difference between caregiving and non-caregiving groups

	Caregivers	Non-caregivers	Full sample	Percentage
	n = 479	n = 2063	n = 2542	difference
	# (%)	# (%)	# (%)	%
Mobility				
No difficulty (1)	221 (46.14)	1470 (71.26)	1691 (66.52)	− 25.12
Slight difficulty (2)	90 (18.79)	257 (12.46)	347 (13.65)	6.33
Some difficulty (3)	121 (25.26)	223 (10.81)	344 (13.53)	14.45
A lot of difficulty (4)	45 (9.39)	106 (5.14)	151 (5.94)	4.25
Unable (5)	2 (0.42)	7 (0.34)	9 (0.35)	0.08
Activities				
No difficulty (1)	179 (37.37)	1201 (58.22)	1380 (54.29)	− 20.85
Slight difficulty (2)	158 (32.99)	468 (22.69)	626 (24.63)	10.3
Some difficulty (3)	88 (18.37)	253 (12.26)	341 (13.41)	6.11
A lot of difficulty (4)	47 (9.81)	121 (5.87)	168 (6.61)	3.94
Unable (5)	7 (1.46)	20 (0.97)	27 (1.06)	0.49
Exhaustion				
None of the time (1)	53 (11.06)	532 (25.79)	585 (23.01)	− 14.73
Only occasionally (2)	172 (35.91)	654 (31.70)	826 (32.49)	4.21
Sometimes (3)	144 (30.06)	460 (22.30)	604 (23.76)	7.76
Often (4)	87 (18.16)	305 (14.78)	392 (15.42)	3.38
Most or all of the time (5)	23 (4.80)	112 (5.43)	135 (5.31)	− 0.63
Loneliness				
None of the time (1)	117 (24.43)	920 (44.60)	1037 (40.79)	− 20.17
Only occasionally (2)	152 (31.73)	485 (23.51)	637 (25.06)	8.22
Sometimes (3)	133 (27.77)	345 (16.72)	478 (18.80)	11.05
Often (4)	60 (12.53)	228 (11.05)	288 (11.33)	1.48
Most or all of the time (5)	17 (3.55)	85 (4.12)	102 (4.01)	− 0.57
Cognition				
None of the time (1)	100 (20.88)	776 (37.62)	876 (34.46)	− 16.74
Only occasionally (2)	177 (36.95)	557 (27.00)	734 (28.87)	9.95
Sometimes (3)	127 (26.51)	439 (21.28)	566 (22.27)	5.23
Often (4)	61 (12.73)	210 (10.18)	271 (10.66)	2.55
Most or all of the time (5)	14 (2.92)	81 (3.93)	95 (3.74)	− 1.01
Anxiety				
None of the time (1)	90 (18.79)	772 (37.42)	862 (33.91)	− 18.63
Only occasionally (2)	155 (32.36)	548 (26.56)	703 (27.66)	5.8
Sometimes (3)	140 (29.23)	386 (18.71)	526 (20.69)	10.52
Often (4)	74 (15.45)	260 (12.60)	334 (13.14)	2.85
Most or all of the time (5)	20 (4.18)	97 (4.70)	117 (4.60)	− 0.52
Sad/depression				
None of the time (1)	109 (22.76)	863 (41.83)	972 (38.24)	− 19.07
Only occasionally (2)	168 (35.07)	538 (26.08)	706 (27.77)	8.99
Sometimes (3)	118 (24.63)	366 (17.74)	484 (19.04)	6.89
Often (4)	63 (13.15)	209 (10.13)	272 (10.70)	3.02
Most or all of the time (5)	21 (4.38)	87 (4.22)	108 (4.25)	0.16
Control				
None of the time (1)	131 (27.35)	990 (47.99)	1121 (44.10)	− 20.64
Only occasionally (2)	162 (33.82)	450 (21.81)	612 (24.08)	12.01
Sometimes (3)	117 (24.43)	356 (17.26)	473 (18.61)	7.17
Often (4)	52 (10.86)	184 (8.92)	236 (9.28)	1.94
Most or all of the time (5)	17 (3.55)	83 (4.02)	100 (3.93)	− 0.47
Pain				
No physical pain (1)	144 (30.06)	839 (40.67)	983 (38.67)	− 10.61
Mild physical pain (2)	185 (38.62)	748 (36.26)	933 (36.70)	2.36
Moderate physical pain (3)	105 (21.92)	347 (16.82)	452 (17.78)	5.1
Severe physical pain (4)	38 (7.93)	107 (5.19)	145 (5.70)	2.74
Very severe physical pain (5)	7 (1.46)	22 (1.07)	29 (1.14)	0.39

### Known group validity analysis by item

We compared mean EQ-HWB-9 item scores between caregiver and non-caregiver samples and found significant differences between groups on all items, as shown in Table 3. For both groups, there were higher mean scores for the psycho-social items 3–8 (exhaustion, loneliness, cognition, anxiety, sad/depressed, control) than the physical items (mobility, activities, and pain). Cohen’s *d* effect size results suggested that differences between groups were largest for the mobility item, and smallest for cognition.

**Table 3:** Known groups validity analysis by Caregiver status by EQ-HWB-9 item

	Caregivers (n = 479)		Non-caregivers (n = 2063)		Mean difference	*t*	*p*-value	Cohen's *d*
	Mean	SD	Mean	SD				
Mobility	1.99	1.06	1.51	0.90	− 0.48	− 10.19	< .001	− 0.52
Activities	2.05	1.04	1.69	0.96	− 0.36	− 7.32	< .001	− 0.37
Exhaustion	2.70	1.04	2.42	1.18	− 0.27	− 4.68	< .001	− 0.24
Loneliness	2.39	1.09	2.07	1.19	− 0.32	− 5.45	< .001	− 0.28
Cognition	2.40	1.04	2.16	1.15	− 0.24	− 4.19	< .001	− 0.21
Anxiety	2.54	1.09	2.21	1.20	− 0.33	− 5.55	< .001	− 0.28
Sad/depression	2.41	1.11	2.09	1.17	− 0.33	− 5.53	< .001	− 0.28
Control	2.29	1.09	1.99	1.17	− 0.30	− 5.16	< .001	− 0.26
Pain	2.12	0.98	1.90	0.93	− 0.22	− 4.69	< .001	− 0.24

Degrees of freedom = 2540 for all tests

*Caregiving for persons with a disability, elderly, dementia, of any age

**Cohen’s *d:* 0.2–0.49 = small, 0.5–0.79 = moderate, above 0.8 = large

### Known group validity analysis by sum- and index scores

Known group validity analyses for EQ-HWB-9 sum- and index scores are presented in Table 4. For the sum-score, there were significant differences between groups in hypothesised directions for all known group validity tests, except for low income. Caregivers, people with mental distress, a chronic health condition, a disability and sleep issues had significantly lower quality-of-life (higher EQ-HWB-9 sum-scores and lower-index scores) than non-caregivers. Effect sizes (Cohen’s *d*) were large (over .8) for mental distress and sleep, and moderate for caregiver status, chronic health condition and disability. Result for the index scores were similar, except that the low-income variable was now statistically significant, with a small effect size. Known-groups analysis by country is displayed in Table S3a.

**Table 4:** Known groups validity analysis results for sum- and index-scores across six variables (df = 2540 for all tests), and comparing female and male Cohen’s *d* effect sizes

Group Membership	Yes			No			Mean difference	*t*	*p*-value	Cohen’s d**
	n	Mean	SD	n	Mean	SD				
EQ-HWB-9 sum-score										
Caregiver*	479	20.90	6.53	2063	18.03	7.34	− 2.87	− 7.86	< .001	− 0.40
Mental health distress	521	27.96	4.92	2021	16.15	5.65	− 11.81	− 43.67	< .001	− 2.15
Chronic health condition	1235	20.39	7.38	1307	16.85	6.76	− 3.54	− 12.64	< .001	− 0.50
Disability	335	22.34	7.68	2207	17.99	7.05	− 4.35	− 10.39	< .001	− 0.61
Sleep issues	1180	22.62	6.91	1362	15.06	5.58	− 7.56	− 30.49	< .001	− 1.21
Low income	671	18.92	7.78	1871	18.44	7.09	− 0.48	− 1.47	.142	− 0.07
EQ-HWB-9 index-score										
Caregiver*	479	0.683	0.217	2063	0.764	0.224	0.081	7.21	< .001	0.37
Mental health distress	521	0.470	0.203	2021	0.820	0.167	0.350	40.81	< .001	2.01
Chronic health condition	1235	0.680	0.240	1307	0.813	0.188	0.133	15.58	< .001	0.62
Disability	335	0.581	.265	2207	0.774	0.206	0.193	15.27	< .001	0.90
Sleep issues	1180	0.631	0.234	1362	0.850	0.157	0.219	28.00	< .001	1.11
Low income	671	0.719	0.253	1871	0.759	0.213	0.040	4.01	< .001	0.18

*Caregiving for persons with a disability and/or long-term illness, and/or dementia and/or problems related to old age and/or elderly family member, compared to all other participants

**Cohen’s *d:* 0.2–0.49 = small, 0.5–0.79 = moderate, above 0.8 = large

^Difference in Cohen’s *d* between female and male participants

### Known group validity analyses by subgroup—gender and age

**Fig. 2:**
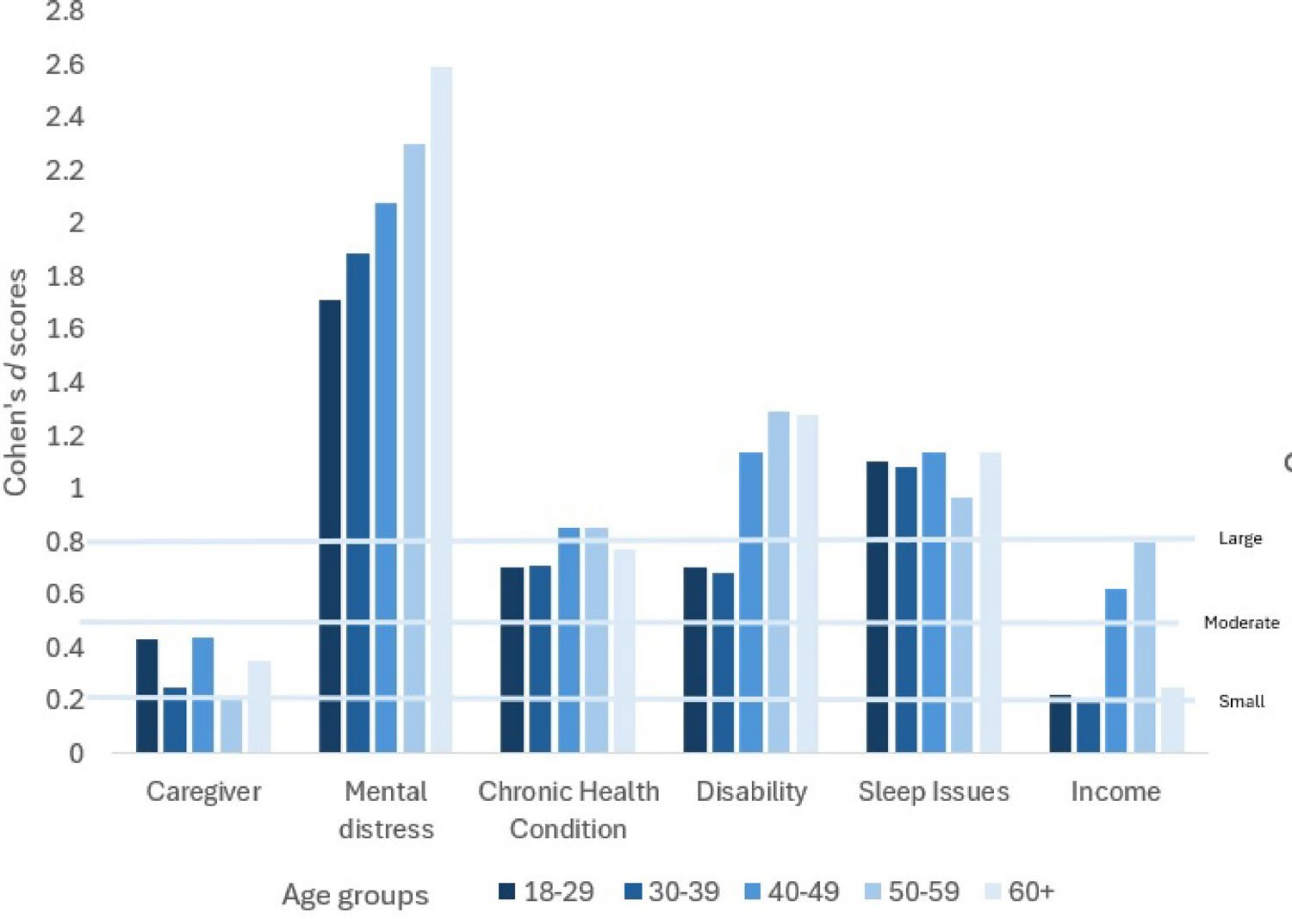
Cohen’s *d* difference related to group membership by age group (EQ-HWB-9 index-scores). Cohen’s *d*: 0.2–0.49 = small, 0.5–0.79 = moderate, above 0.8 = large

Known group validity test results by gender are presented in Table S3b. Differences between female and male Cohen’s *d* scores were minimal. Known group validity test results by age group are presented in Table S3c. Cohen’s *d* scores by age group are displayed in Fig. 2. There were very large Cohen’s *d* scores for mental distress, and increased scores by age. There were large differences between the 18–39 age groups and the 40+ ages for disability, with high Cohen’s *d* scores for the older age groups, and moderate for the younger. Caregiver status, chronic health condition and sleep issues did not appear to have a strong association with age. Cohen’s *d* scores were highest for the 40–59-year age bracket for low income.

### Convergent/divergent validity analysis

We assessed convergent validity using Spearman correlations between the EQ-HWB-9 and the K6 questionnaire items, the K6 total score and the EQ-HWB-9 sum- and index-scores, as shown in Table 5. Items hypothesised to be at least moderately correlated are bolded. None of the correlations between the EQ-HWB-9 pain item and the K6 items were above .3, and mobility and activities were only weakly or less correlated with K6 items. The correlation between the EQ-HWB-9 sum-score and K6 total score was over .85 and slightly lower for the index score at .79. All our hypotheses were supported. Analyses by country are presented in Tables S4a and S4b. Results were consistent across countries.

**Table 5:**
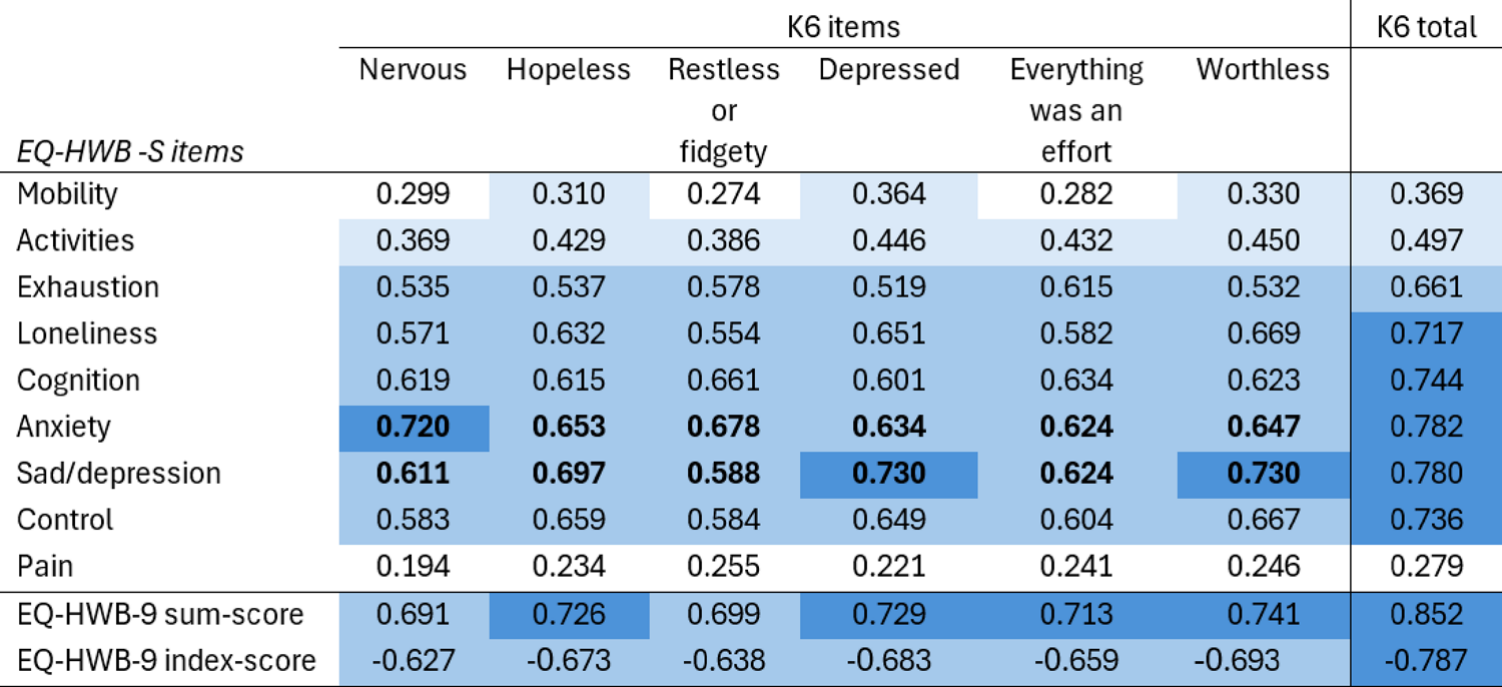
Convergent validity

All correlations are significant at *p* < .001

Cohen’s *d*: 0.2-0.49= small (coded light blue), 0.5-0.79 = moderate (coded medium blue), above 0.8 = large (coded dark blue)

Items hypothesised to be at least moderately correlated are bolded within the matrix.

## Discussion

We aimed to assess the validity of the EQ-HWB-9 in a general population sample of the Australian and New Zealand populations. We found differences in baseline characteristics between caregiver and non-caregiver groups for age, employment, LGBTQIA+ status, mental health distress, chronic health condition and sleep problems, but not for gender, household composition, disability status or indigenous status. Our finding of a relatively even gender split is consistent with ABS data where 12.8% of all females and 11.1% of all males identified as informal carers in 2022, of which females were much more likely to identify as the primary carer [30].

Similarly to our previous work, we found that EQ-HWB-9 item responses were well distributed, and that participants scored higher on the psycho-social items than the physical items [11, 12]. When comparing the distribution for caregivers to non-caregivers, caregivers had significantly worse mean scores for each of the EQ-HWB-9 items compared to their counterparts. This result is similar to our previous study of caregivers of children with health conditions compared to a general population sample of parents [11], which found that parents of children with health conditions had lower scores over all items. Differences in demographic characteristics of caregivers across countries may have driven the differences found in item distributions for caregivers in New Zealand compared to Australia.

Our hypotheses were supported for the known group validity analyses comparing caregiver, mental health distress, chronic health condition, disability, and sleep issues variables. We found minimal differences between males and females when comparing effect sizes across gender, as we had also found in our previous study of parents of children with health conditions compared to the general population [11]. We note that some of the effect-sizes were higher for males than females (for instance, caregiver status and low income) which seem counter-intuitive given what we know of the increased burden of caregiving on women (although differences were small here). It appeared that there were higher impacts on quality-of-life for males when experiencing low income, but it is not possible to determine why this might be the case from the available information.

We had hypothesised that people with low income would have lower quality-of-life; this hypothesis was supported when preference-weights were applied, but not when using the sum-score. The difference in Cohen’s *d* scores between the sum- and index-scores demonstrated the effect of the preference-weighting on the data. The pilot preference-weights give higher preferences to the domains of mobility, activities and pain (i.e. the physical items), and downplay the psycho-social items, in particular exhaustion, cognition, anxiety, and control, and are not specific to Australia or New Zealand. Results were similar for Australia (noting the larger sample size), but for New Zealand, there were no significant differences between groups for caregivers, but low income was significant; it is unclear why there would be country differences on these variables, but this outcome highlights the importance of also considering the data by country.

When we investigated the effect of age-group on Cohen’s *d* scores, we found that Cohen’s *d* scores increased as age increased for the mental health variable, suggesting that quality-of-life differences between people with and without mental distress (as per the published cut-points for the K6) increased with age. We also saw a jump in Cohen’s *d* scores for disability for the 40 + groups, suggesting that the differences in quality-of-life between people with and without a disability increase after the age of 40. Age group differences were not pronounced for caregiver status, chronic health condition or sleep, suggesting that it was not necessary to control for age in the analyses.

When looking at the effect size differences by item, comparing caregiver scores to their counterparts, Cohen’s *d* effect sizes and mean differences were largest for mobility and smallest for cognition. In our previous work, where we compared parents of children with a health condition to the general population, we found a different pattern in which the lowest mean differences were for mobility, and the highest for exhaustion and cognition [11]. It is possible that these differences are due to the different samples; in the current study we did not include caregivers of children (with no other caregiver duties) in our caregiver group, whereas in our previous study all participants were parents, with and without a child with a health condition [11].

Results from the convergent validity analysis comparing the EQ-HWB-9 items with the K6 were similar to our previous study of caregivers of children aged eight or less where families had experienced adversity [12]. In both studies, mobility, activities and pain had the lowest correlations with the K6 items, and both studies conformed to a priori hypotheses. Comparing the EQ-HWB-9 to other relevant instruments would be a next step in validating the EQ-HWB-9 prior to future release.

### Strengths and limitations

A major strength of this study was the use of a large general population dataset from two countries, nationally representative on age, gender, region, ancestry/ethnicity, and income, which gave us a sufficient sample size to conduct psychometric testing by country, gender and age subgroups. For our caregiving variable, we included those participants caring for a person with a disability, long-term illness, dementia and/or problems related to old age, and/or an elderly family member, thus enabling us to only include those participants where we hypothesised there would be a significant caregiver effect. As this was a secondary analysis, comparison instruments were limited to the K6.

We used the pilot UK value-set and note that the preference-weights were not specific for Australia or New Zealand; this may have affected outcomes. This value-set was developed to test the method for producing preference-weights and is therefore only indicative of possible index values. Country-specific preference-weights will be produced when the instrument is finally released [31]. The level sum-score has known limitations as it gives equal weight to all items [32]; however, recent research has suggested that the EQ-HWB-9 items on their own form a strong instrument with strong psychosocial and physical sub-domains, suggesting that using an un-weighted approach may be a promising alternative [33]. The differences we found in sum- and index-score results suggest that more investigation into these methods is required.

Due to being a secondary analysis, we were unable to collect more detailed information on caregiving, such as time spent caregiving, caregiving intensity or primary caregiver status, as we had in our previous research [34]. We observed a higher prevalence of informal carers (18.8%) compared to national estimates of 11% [35], and higher identification with LGBTQIA+ individuals (14%) compared to national estimates of 4.5% [36]. We also found high levels of chronic illness and mobility concerns in the caregiver group compared to the non-caregiver group, which may be confounding factors in the known-group differences analysis. We cannot rule out sample bias given that online panels may over-represent younger, progressive or “survey engaged” individuals, which could inflate those specific prevalence estimates. We note that our LGBTQIA+ question was more inclusive of sexuality and gender diversity than questions asked by other data sources which have focused on sexual identity alone (ABS [36] and HILDA [37]);thus, it is equally possible that our findings reflect better measurement rather than sampling error. We note that our results more closely matched those of younger people who report higher LGBTQIA+ rates, with 42.5% of secondary school students in an Australian sample identifying as LGBTQIA+  [38]. We also found high rates of LGBTQIA+ in caregivers, possibly due to a higher likelihood of caring for LGBTQIA+ friends and chosen family in addition to biological family members [39]. Further research on using the EQ-HWB instruments in these populations is warranted.

## Conclusion

This study adds to increasingly strong evidence supporting the use of the EQ-HWB-9 as a suitable instrument for measuring general population and caregiver health and wellbeing in a range of settings, and for economic analysis. Our previous work has been in caregiver populations; this study, using a broad population sample as well as including caregivers, can be considered as generalizable across a range of populations. The data was evenly distributed across response options for most items and showed strong known-group and convergent validity. Known-group validity testing was conducted at the item, sum-score and index-score levels, as well as by country, gender and age. Further research to explore differences between the sum-score and index-scores is warranted. Such work would also provide methods for testing future preference-weighted value-sets as they are produced.

## Supplementary Information

Below is the link to the electronic supplementary material.

Supplementary file1 (DOCX 129 kb)

## Data Availability

Data, code and other materials related to this study will be made available on the Open Science Framework at osf.io/4jzwf/
