# Supplementary material for: The psychometric performance of the EQ-HWB-9 for measuring health and wellbeing in a general population sample from Australia and New Zealand
Source: Qual Life Res. 2025 Sep 18;34(12):3707–19. doi: 10.1007/s11136-025-04061-3 (PMC12689816; doi:10.1007/s11136-025-04061-3)
Supplement: Supplementary file 1 — Supplementary file1 (DOCX 129 kb) [file 11136_2025_4061_MOESM1_ESM.docx]

**The psychometric performance of the EQ-HWB-S for measuring health and wellbeing in caregivers using nationally representative samples of the Australia and New Zealand population**

# Table S1a – Baseline – Australia

| **Variable** |  | **Caregiver*** | **Non-caretivers** | **Total** |  |  |
| --- | --- | --- | --- | --- | --- | --- |
|  |  | (n=423) | (n=1595) | (n=2018) |  |  |
|  |  | **# (%)** | **# (%)** | **# (%)** | **chi2 (df)** | **p-value** |
| **Sex** |  |  |  |  | **0.76 (2)** | **0.685** |
|  | Female | 212 (50.12) | 831 (52.10) | 1043 (51.68) |  |  |
|  | Male | 210 (49.65) | 758 (47.52) | 968 (47.97) |  |  |
|  | Other (All) | 1 (0.24) | 6 (0.38) | 7 (0.35) |  |  |
| **Education** |  |  |  |  | **26.93 (7)** | **< 0.001** |
|  | Less than Primary | 1 (0.24) | 3 (0.19) | 4 (0.20) |  |  |
|  | Primary | 1 (0.24) | 8 (0.50) | 9 (0.45) |  |  |
|  | Some Secondary | 18 (4.26) | 143 (8.97) | 161 (7.98) |  |  |
|  | Secondary | 59 (13.95) | 291 (18.24) | 350 (17.34) |  |  |
|  | Vocational or Similar | 89 (21.04) | 342 (21.44) | 431 (21.36) |  |  |
|  | Some University but no degree | 74 (17.49) | 171 (10.72) | 245 (12.14) |  |  |
|  | University - Bachelor’s Degree | 136 (32.15) | 488 (30.60) | 624 (30.92) |  |  |
|  | Graduate or professional degree | 45 (10.64) | 149 (9.34) | 194 (9.61) |  |  |
| **Employment** |  |  |  |  | **70.42 (6)** | **< 0.001** |
|  | Working full-time | 285 (67.38) | 718 (45.02) | 1003 (49.70) |  |  |
|  | Working part-time | 56 (13.24) | 289 (18.12) | 345 (17.10) |  |  |
|  | Studying | 6 (1.42) | 45 (2.82) | 51 (2.53) |  |  |
|  | Unemployed and looking for work | 12 (2.84) | 74 (4.64) | 86 (4.26) |  |  |
|  | Unemployed and not looking for work | 11 (2.60) | 65 (4.08) | 76 (3.77) |  |  |
|  | Retired | 44 (10.40) | 358 (22.45) | 402 (19.92) |  |  |
|  | Other | 9 (2.13) | 46 (2.88) | 55 (2.73) |  |  |
| **Household** |  |  |  |  | **2.81 (4)** | **0.590** |
|  | Alone | 97 (22.93) | 351 (22.01) | 448 (22.20) |  |  |
|  | Single parent | 49 (11.58) | 175 (10.97) | 224 (11.10) |  |  |
|  | Couple with children | 104 (24.59) | 349 (21.88) | 453 (22.45) |  |  |
|  | Couple | 118 (27.90) | 501 (31.41) | 619 (30.67) |  |  |
|  | other | 55 (13.00) | 219 (13.73) | 274 (13.58) |  |  |
| **Identifies as LGBT** |  |  |  |  | **155.51 (2)** | **< 0.001** |
|  | No | 277 (65.48) | 1428 (89.53) | 1705 (84.49) |  |  |
|  | Yes | 144 (34.04) | 156 (9.78) | 300 (14.87) |  |  |
|  | Prefer not to say | 2 (0.47) | 11 (0.69) | 13 (0.64) |  |  |
| **Mental health distress** | |  |  |  | **27.03 (1)** | **< 0.001** |
|  | No | 292 (69.03) | 1288 (80.75) | 1580 (78.30) |  |  |
|  | Yes | 131 (30.97) | 307 (19.25) | 438 (21.70) |  |  |
| **Chronic health condition** | |  |  |  | **72.34 (1)** | **< 0.001** |
|  | No | 135 (31.91) | 880 (55.17) | 1015 (50.30) |  |  |
|  | Yes | 288 (68.09) | 715 (44.83) | 1003 (49.70) |  |  |
| **Disability** |  |  |  |  | **0.02 (1)** | **0.892** |
|  | No or Prefer not to say | 371 (87.71) | 1395 (87.46) | 1766 (87.51) |  |  |
|  | Yes | 52 (12.29) | 200 (12.54) | 252 (12.49) |  |  |
| **Sleep issues** |  |  |  |  | **35.98 (1)** | **< 0.001** |
|  | None of the time or Only Occasionally | 167 (39.48) | 891 (55.86) | 1058 (52.43) |  |  |
|  | Sometimes, Often, Most or all of the time | 256 (60.52) | 704 (44.14) | 960 (47.57) |  |  |
| **Income** |  |  |  |  | **16.37 (1)** | **< 0.001** |
|  | Other Income | 349 (82.51) | 1163 (72.92) | 1512 (74.93) |  |  |
|  | Low Income | 74 (17.49) | 432 (27.08) | 506 (25.07) |  |  |
| **Indigenous status (AU)** | |  |  |  | **0.32 (1)** | **0.573** |
|  | Not | 408 (96.45) | 1547 (96.99) | 1955 (96.88) |  |  |
|  | ATSI | 15 (3.55) | 48 (3.01) | 63 (3.12) |  |  |
|  |  | **Mean (SD)** | **Mean (SD)** | **Mean (SD)** | **t (df)** | **p-value** |
| **K6 total score** | | 15.19 (5.28) | 12.95 (5.92) | 13.42 (5.86) | **-7.06 (2016)** | **< 0.001** |
| **Age (continuous)** | | 43.76 (15.27) | 47.66 (18.3) | 46.84 (17.78) | **4.03 (2016)** | **< 0.001** |

# Table S1b – Baseline – New Zealand

| **Variable** |  | **Caregiver*** | **Non-caregivers** | **Total** |  |  |
| --- | --- | --- | --- | --- | --- | --- |
|  |  | (n=56) | (n=468) | (n=524) |  |  |
|  |  | **# (%)** | **# (%)** | **# (%)** | **chi2 (df)** | **p-value** |
| **Sex** |  |  |  |  | **0.43 (2)** | **0.807** |
|  | Female | 28 (50.00) | 241 (51.50) | 269 (51.34) |  |  |
|  | Male | 28 (50.00) | 224 (47.86) | 252 (48.09) |  |  |
|  | Other (All) | 0 (0.00) | 3 (0.64) | 3 (0.57) |  |  |
| **Education** |  |  |  |  | **2.69 (6)** | **0.847** |
|  | Primary | 1 (1.79) | 2 (0.43) | 3 (0.57) |  |  |
|  | Some Secondary | 5 (8.93) | 37 (7.91) | 42 (8.02) |  |  |
|  | Secondary | 10 (17.86) | 96 (20.51) | 106 (20.23) |  |  |
|  | Vocational or Similar | 11 (19.64) | 98 (20.94) | 109 (20.80) |  |  |
|  | Some University but no degree | 9 (16.07) | 59 (12.61) | 68 (12.98) |  |  |
|  | University - Bachelors Degree | 14 (25.00) | 133 (28.42) | 147 (28.05) |  |  |
|  | Graduate or professional degree | 6 (10.71) | 43 (9.19) | 49 (9.35) |  |  |
| **Employment** |  |  |  |  | **3.43 (6)** | **0.753** |
|  | Working full-time | 27 (48.21) | 248 (52.99) | 275 (52.48) |  |  |
|  | Working part-time | 10 (17.86) | 80 (17.09) | 90 (17.18) |  |  |
|  | Studying | 2 (3.57) | 17 (3.63) | 19 (3.63) |  |  |
|  | Unemployed and looking for work | 3 (5.36) | 25 (5.34) | 28 (5.34) |  |  |
|  | Unemployed and not looking for work | 2 (3.57) | 18 (3.85) | 20 (3.82) |  |  |
|  | Retired | 9 (16.07) | 72 (15.38) | 81 (15.46) |  |  |
|  | Other | 3 (5.36) | 8 (1.71) | 11 (2.10) |  |  |
| **Household** |  |  |  |  | **4.17 (4)** | **0.383** |
|  | Alone | 8 (14.29) | 85 (18.16) | 93 (17.75) |  |  |
|  | Single parent | 4 (7.14) | 41 (8.76) | 45 (8.59) |  |  |
|  | Couple with children | 8 (14.29) | 107 (22.86) | 115 (21.95) |  |  |
|  | Couple | 26 (46.43) | 171 (36.54) | 197 (37.60) |  |  |
|  | other | 10 (17.86) | 64 (13.68) | 74 (14.12) |  |  |
| **Identifies as LGBT** |  |  |  |  | **1.11 (2)** | **0.575** |
|  | No | 48 (85.71) | 414 (88.46) | 462 (88.17) |  |  |
|  | Yes | 8 (14.29) | 50 (10.68) | 58 (11.07) |  |  |
|  | Prefer not to say | 0 (0.00) | 4 (0.85) | 4 (0.76) |  |  |
| **Mental health distress** |  |  |  |  | **1.47 (1)** | **0.225** |
|  | No | 44 (78.57) | 397 (84.83) | 441 (84.16) |  |  |
|  | Yes | 12 (21.43) | 71 (15.17) | 83 (15.84) |  |  |
| **Chronic health condition** |  |  |  |  | **1.43 (1)** | **0.231** |
|  | No | 27 (48.21) | 265 (56.62) | 292 (55.73) |  |  |
|  | Yes | 29 (51.79) | 203 (43.38) | 232 (44.27) |  |  |
| **Disability** |  |  |  |  | **0.68 (1)** | **0.409** |
|  | No or Prefer not to say | 45 (80.36) | 396 (84.62) | 441 (84.16) |  |  |
|  | Yes | 11 (19.64) | 72 (15.38) | 83 (15.84) |  |  |
| **Sleep issues** |  |  |  |  | **0.51 (1)** | **0.476** |
|  | None of the time or Only Occassionally | 30 (53.57) | 274 (58.55) | 304 (58.02) |  |  |
|  | Sometimes, Often, Most or all of the time | 26 (46.43) | 194 (41.45) | 220 (41.98) |  |  |
| **Income** |  |  |  |  | **5.03 (1)** | **0.025** |
|  | Other Income | 31 (55.36) | 328 (70.09) | 359 (68.51) |  |  |
|  | Low Income | 25 (44.64) | 140 (29.91) | 165 (31.49) |  |  |
| **Indigenous status (NZ)** |  |  |  |  | **6.14 (4)** | **0.189** |
|  | Non-Indigenous | 39 (69.64) | 358 (76.50) | 397 (75.76) |  |  |
|  | Māori | 14 (25.00) | 94 (20.09) | 108 (20.61) |  |  |
|  | Cook Islands Māori | 1 (1.79) | 7 (1.50) | 8 (1.53) |  |  |
|  | Niuean | 0 (0.00) | 6 (1.28) | 6 (1.15) |  |  |
|  | Multiple | 2 (3.57) | 3 (0.64) | 5 (0.95) |  |  |
|  |  | **Mean (SD)** | **Mean (SD)** | **Mean (SD)** | **t (df)** | **p-value** |
| **K6 total score** |  | 13.36 (5.53) | 12.79 (5.66) | 12.85 (5.64) | **-0.71 (522)** | **0.478** |
| **Age (continuous)** |  | 46.55 (17.56) | 46.29 (17.93) | 46.32 (17.88) | **-0.10 (522)** | **0.919** |

# Table S2a – Distribution of EQ-HWB-S scores by caregiver status - Australia

|  | | | |  |  |
| --- | --- | --- | --- | --- | --- |
|  |  | **Caregivers** | **Non-caregivers** | **Full sample** | **Percentage** |
|  |  | **n= 423** | **n= 1595** | **n= 2018** | **difference*** |
| Mobility |  | **# (%)** | **# (%)** | **# (%)** | **%** |
|  | No difficulty | 181 (42.79) | 1118 (70.09) | 1299 (64.37 ) | -27.3 |
|  | Slight difficulty | 81 (19.15) | 194 (12.16) | 275 (13.63 ) | 6.99 |
|  | Some difficulty | 117 (27.66) | 181 (11.35) | 298 (14.77 ) | 16.31 |
|  | A lot of difficulty | 42 (9.93) | 96 (6.02) | 138 (6.84 ) | 3.91 |
|  | Unable | 2 (0.47) | 6 (0.38) | 8 (0.40 ) | 0.09 |
| Activities |  |  |  |  |  |
|  | No difficulty | 149 (35.22) | 914 (57.30) | 1063 (52.68 ) | -22.08 |
|  | Slight difficulty | 143 (33.81) | 365 (22.88) | 508 (25.17 ) | 10.93 |
|  | Some difficulty | 82 (19.39) | 198 (12.41) | 280 (13.88 ) | 6.98 |
|  | A lot of difficulty | 42 (9.93) | 101 (6.33) | 143 (7.09 ) | 3.6 |
|  | Unable | 7 (1.65) | 17 (1.07) | 24 (1.19 ) | 0.58 |
| Exhaustion | |  |  |  |  |
|  | None of the time | 46 (10.87) | 424 (26.58) | 470 (23.29 ) | -15.71 |
|  | Only occasionally | 149 (35.22) | 494 (30.97) | 643 (31.86 ) | 4.25 |
|  | Sometimes | 131 (30.97) | 360 (22.57) | 491 (24.33 ) | 8.4 |
|  | Often | 79 (18.68) | 234 (14.67) | 313 (15.51 ) | 4.01 |
|  | Most or all of the time | 18 (4.26) | 83 (5.20) | 101 (5.00 ) | -0.94 |
| Loneliness |  |  |  |  |  |
|  | None of the time | 97 (22.93) | 698 (43.76) | 795 (39.40 ) | -20.83 |
|  | Only occasionally | 141 (33.33) | 373 (23.39) | 514 (25.47 ) | 9.94 |
|  | Sometimes | 121 (28.61) | 275 (17.24) | 396 (19.62 ) | 11.37 |
|  | Often | 51 (12.06) | 185 (11.60) | 236 (11.69 ) | 0.46 |
|  | Most or all of the time | 13 (3.07) | 64 (4.01) | 77 (3.82 ) | -0.94 |
| Cognition |  |  |  |  |  |
|  | None of the time | 81 (19.15) | 599 (37.55) | 680 (33.70 ) | -18.4 |
|  | Only occasionally | 162 (38.30) | 424 (26.58) | 586 (29.04 ) | 11.72 |
|  | Sometimes | 115 (27.19) | 341 (21.38) | 456 (22.60 ) | 5.81 |
|  | Often | 54 (12.77) | 169 (10.60) | 223 (11.05 ) | 2.17 |
|  | Most or all of the time | 11 (2.60) | 62 (3.89) | 73 (3.62 ) | -1.29 |
| Anxiety |  |  |  |  |  |
|  | None of the time | 73 (17.26) | 585 (36.68) | 658 (32.61 ) | -19.42 |
|  | Only occasionally | 138 (32.62) | 425 (26.65) | 563 (27.90 ) | 5.97 |
|  | Sometimes | 130 (30.73) | 306 (19.18) | 436 (21.61 ) | 11.55 |
|  | Often | 66 (15.60) | 210 (13.17) | 276 (13.68 ) | 2.43 |
|  | Most or all of the time | 16 (3.78) | 69 (4.33) | 85 (4.21 ) | -0.55 |
| Sad/depression | |  |  |  |  |
|  | None of the time | 90 (21.28) | 645 (40.44) | 735 (36.42 ) | -19.16 |
|  | Only occasionally | 150 (35.46) | 415 (26.02) | 565 (28.00 ) | 9.44 |
|  | Sometimes | 113 (26.71) | 298 (18.68) | 411 (20.37 ) | 8.03 |
|  | Often | 50 (11.82) | 169 (10.60) | 219 (10.85 ) | 1.22 |
|  | Most or all of the time | 20 (4.73) | 68 (4.26) | 88 (4.36 ) | 0.47 |
| Control |  |  |  |  |  |
|  | None of the time | 108 (25.53) | 747 (46.83) | 855 (42.37 ) | -21.3 |
|  | Only occasionally | 146 (34.52) | 358 (22.45) | 504 (24.98 ) | 12.07 |
|  | Sometimes | 107 (25.30) | 287 (17.99) | 394 (19.52 ) | 7.31 |
|  | Often | 47 (11.11) | 137 (8.59) | 184 (9.12 ) | 2.52 |
|  | Most or all of the time | 15 (3.55) | 66 (4.14) | 81 (4.01 ) | -0.59 |
| Pain |  |  |  |  |  |
|  | No physical pain | 127 (30.02) | 655 (41.07) | 782 (38.75 ) | -11.05 |
|  | Mild physical pain | 160 (37.83) | 578 (36.24) | 738 (36.57 ) | 1.59 |
|  | Moderate physical pain | 97 (22.93) | 268 (16.80) | 365 (18.09 ) | 6.13 |
|  | Severe physical pain | 33 (7.80) | 76 (4.76) | 109 (5.40 ) | 3.04 |
|  | Very severe physical pain | 6 (1.42) | 18 (1.13) | 24 (1.19 ) | 0.29 |
|  |  |  |  |  |  |

# Table S2b – Distribution of EQ-HWB-S scores by caregiver status – New Zealand

|  | | | |  |  |
| --- | --- | --- | --- | --- | --- |
|  |  | **Caregivers** | **Non-caregivers** | **Full sample** | **Percentage** |
|  |  | **n= 56** | **n= 468** | **n= 524** | **difference*** |
| Mobility |  | **# (%)** | **# (%)** | **# (%)** | **%** |
|  | No difficulty | 40 (71.43) | 352 (75.21) | 392 (74.81) | -3.78 |
|  | Slight difficulty | 9 (16.07) | 63 (13.46) | 72 (13.74) | 2.61 |
|  | Some difficulty | 4 (7.14) | 42 (8.97) | 46 (8.78) | -1.83 |
|  | A lot of difficulty | 3 (5.36) | 10 (2.14) | 13 (2.48) | 3.22 |
|  | Unable | 0 (0.00) | 1 (0.21) | 1 (0.19) | -0.21 |
| Activities |  |  |  |  |  |
|  | No difficulty | 30 (53.57) | 287 (61.32) | 317 (60.50) | -7.75 |
|  | Slight difficulty | 15 (26.79) | 103 (22.01) | 118 (22.52) | 4.78 |
|  | Some difficulty | 6 (10.71) | 55 (11.75) | 61 (11.64) | -1.04 |
|  | A lot of difficulty | 5 (8.93) | 20 (4.27) | 25 (4.77) | 4.66 |
|  | Unable | 0 (0.00) | 3 (0.64) | 3 (0.57) | -0.64 |
| Exhaustion |  |  |  |  |  |
|  | None of the time | 7 (12.50) | 108 (23.08) | 115 (21.95) | -10.58 |
|  | Only occasionally | 23 (41.07) | 160 (34.19) | 183 (34.92) | 6.88 |
|  | Sometimes | 13 (23.21) | 100 (21.37) | 113 (21.56) | 1.84 |
|  | Often | 8 (14.29) | 71 (15.17) | 79 (15.08) | -0.88 |
|  | Most or all of the time | 5 (8.93) | 29 (6.20) | 34 (6.49) | 2.73 |
| Loneliness |  |  |  |  |  |
|  | None of the time | 20 (35.71) | 222 (47.44) | 242 (46.18) | -11.73 |
|  | Only occasionally | 11 (19.64) | 112 (23.93) | 123 (23.47) | -4.29 |
|  | Sometimes | 12 (21.43) | 70 (14.96) | 82 (15.65) | 6.47 |
|  | Often | 9 (16.07) | 43 (9.19) | 52 (9.92) | 6.88 |
|  | Most or all of the time | 4 (7.14) | 21 (4.49) | 25 (4.77) | 2.65 |
| Cognition |  |  |  |  |  |
|  | None of the time | 19 (33.93) | 177 (37.82) | 196 (37.40) | -3.89 |
|  | Only occasionally | 15 (26.79) | 133 (28.42) | 148 (28.24) | -1.63 |
|  | Sometimes | 12 (21.43) | 98 (20.94) | 110 (20.99) | 0.49 |
|  | Often | 7 (12.50) | 41 (8.76) | 48 (9.16) | 3.74 |
|  | Most or all of the time | 3 (5.36) | 19 (4.06) | 22 (4.20) | 1.3 |
| Anxiety |  |  |  |  |  |
|  | None of the time | 17 (30.36) | 187 (39.96) | 204 (38.93) | -9.6 |
|  | Only occasionally | 17 (30.36) | 123 (26.28) | 140 (26.72) | 4.08 |
|  | Sometimes | 10 (17.86) | 80 (17.09) | 90 (17.18) | 0.77 |
|  | Often | 8 (14.29) | 50 (10.68) | 58 (11.07) | 3.61 |
|  | Most or all of the time | 4 (7.14) | 28 (5.98) | 32 (6.11) | 1.16 |
| Sad/depression |  |  |  |  |  |
|  | None of the time | 19 (33.93) | 218 (46.58) | 237 (45.23) | -12.65 |
|  | Only occasionally | 18 (32.14) | 123 (26.28) | 141 (26.91) | 5.86 |
|  | Sometimes | 5 (8.93) | 68 (14.53) | 73 (13.93) | -5.6 |
|  | Often | 13 (23.21) | 40 (8.55) | 53 (10.11) | 14.66 |
|  | Most or all of the time | 1 (1.79) | 19 (4.06) | 20 (3.82) | -2.27 |
| Control |  |  |  |  |  |
|  | None of the time | 23 (41.07) | 243 (51.92) | 266 (50.76) | -10.85 |
|  | Only occasionally | 16 (28.57) | 92 (19.66) | 108 (20.61) | 8.91 |
|  | Sometimes | 10 (17.86) | 69 (14.74) | 79 (15.08) | 3.12 |
|  | Often | 5 (8.93) | 47 (10.04) | 52 (9.92) | -1.11 |
|  | Most or all of the time | 2 (3.57) | 17 (3.63) | 19 (3.63) | -0.06 |
| Pain |  |  |  |  |  |
|  | No physical pain | 17 (30.36) | 184 (39.32) | 201 (38.36) | -8.96 |
|  | Mild physical pain | 25 (44.64) | 170 (36.32) | 195 (37.21) | 8.32 |
|  | Moderate physical pain | 8 (14.29) | 79 (16.88) | 87 (16.60) | -2.59 |
|  | Severe physical pain | 5 (8.93) | 31 (6.62) | 36 (6.87) | 2.31 |
|  | Very severe physical pain | 1 (1.79) | 4 (0.85) | 5 (0.95) | 0.94 |

# Table S3a - Known groups validity analysis by country

| Table 4a: Known groups validity analysis - Australia | | |  |  |  |  |  |  |  |  |  |  |
| --- | --- | --- | --- | --- | --- | --- | --- | --- | --- | --- | --- | --- |
| **AUSTRALIA** | **Yes** | | |  | **No** | | | **Mean** |  |  | **Cohen's** |  |
|  | **n** | **Mean** | **SD** |  | **n** | **Mean** | **SD** | **difference** | ***t*** | ***p*-value** | ***d*** | **df** |
| **EQ-HWB-S sum-score** |  |  |  |  |  |  |  |  |  |  |  |  |
| Caregiver | 423 | 21.104 | 6.4 |  | 1595 | 18.152 | 7.444 | -2.952 | -7.46 | < 0.001 | -0.41 | 2016 |
| Mental health distress | 438 | 27.902 | 4.917 |  | 1580 | 16.239 | 5.701 | -11.663 | -38.98 | < 0.001 | -2.1 | 2016 |
| Chronic health condition | 1003 | 20.612 | 7.401 |  | 1015 | 16.951 | 6.8 | -3.661 | -11.57 | < 0.001 | -0.52 | 2016 |
| Disability | 252 | 22.667 | 7.631 |  | 1766 | 18.215 | 7.123 | -4.452 | -9.2 | < 0.001 | -0.62 | 2016 |
| Sleep issues | 960 | 22.692 | 6.953 |  | 1058 | 15.213 | 5.673 | -7.479 | -26.57 | < 0.001 | -1.18 | 2016 |
| Low income | 506 | 18.787 | 7.886 |  | 1512 | 18.765 | 7.145 | -0.022 | -0.06 | 0.955 | 0 | 2016 |
| **EQ-HWB-S index-score** |  |  |  |  |  |  |  |  |  |  |  |  |
| Caregiver | 423 | 0.678 | 0.213 |  | 1595 | 0.76 | 0.228 | 0.082 | 6.7 | < 0.001 | 0.37 | 2016 |
| Mental health distress | 438 | 0.47 | 0.203 |  | 1580 | 0.819 | 0.168 | 0.349 | 36.66 | < 0.001 | 1.98 | 2016 |
| Chronic health condition | 1003 | 0.675 | 0.241 |  | 1015 | 0.81 | 0.19 | 0.135 | 14.01 | < 0.001 | 0.62 | 2016 |
| Disability | 252 | 0.571 | 0.262 |  | 1766 | 0.768 | 0.211 | 0.197 | 13.4 | < 0.001 | 0.90 | 2016 |
| Sleep issues | 960 | 0.629 | 0.235 |  | 1058 | 0.847 | 0.16 | 0.218 | 24.61 | < 0.001 | 1.10 | 2016 |
| Low income | 506 | 0.723 | 0.256 |  | 1512 | 0.75 | 0.216 | 0.027 | 2.37 | 0.018 | 0.12 | 2016 |
|  |  |  |  |  |  |  |  |  |  |  |  |  |

Cohen’s *d:* 0.2-0.49= small, 0.5-0.79 = moderate, above 0.8 = large

| Table 4b: Known groups validity analysis - New Zealand | | | |  |  |  |  |  |  |  |  |  |
| --- | --- | --- | --- | --- | --- | --- | --- | --- | --- | --- | --- | --- |
| **NEW ZEALAND** | **Yes** | | |  | **No** | | | **Mean** |  |  | **Cohen's** |  |
|  | **n** | **Mean** | **SD** |  | **n** | **Mean** | **SD** | **difference** | ***t*** | ***p*-value** | ***d*** | **df** |
| **EQ-HWB-S sum-score** |  |  |  |  |  |  |  |  |  |  |  |  |
| Caregiver | 56 | 19.321 | 7.299 |  | 468 | 17.598 | 6.979 | -1.723 | -1.74 | 0.083 | -0.25 | 522 |
| Mental health distress | 83 | 28.253 | 4.975 |  | 441 | 15.812 | 5.432 | -12.441 | -19.39 | < 0.001 | -2.32 | 522 |
| Chronic health condition | 232 | 19.427 | 7.208 |  | 292 | 16.476 | 6.605 | -2.951 | -4.88 | < 0.001 | -0.43 | 522 |
| Disability | 83 | 21.349 | 7.781 |  | 441 | 17.111 | 6.675 | -4.238 | -5.16 | < 0.001 | -0.62 | 522 |
| Sleep issues | 220 | 22.286 | 6.733 |  | 304 | 14.523 | 5.203 | -7.763 | -14.88 | < 0.001 | -1.32 | 522 |
| Low income | 165 | 19.333 | 7.449 |  | 359 | 17.07 | 6.715 | -2.263 | -3.46 | 0.001 | -0.33 | 522 |
| **EQ-HWB-S index-score** |  |  |  |  |  |  |  |  |  |  |  |  |
| Caregiver | 56 | 0.717 | 0.239 |  | 468 | 0.776 | 0.21 | 0.059 | 1.95 | 0.052 | 0.28 | 522 |
| Mental health distress | 83 | 0.47 | 0.203 |  | 441 | 0.826 | 0.162 | 0.356 | 17.58 | < 0.001 | 2.10 | 522 |
| Chronic health condition | 232 | 0.703 | 0.234 |  | 292 | 0.823 | 0.179 | 0.12 | 6.6 | < 0.001 | 0.58 | 522 |
| Disability | 83 | 0.612 | 0.274 |  | 441 | 0.799 | 0.186 | 0.187 | 7.72 | < 0.001 | 0.92 | 522 |
| Sleep issues | 220 | 0.644 | 0.226 |  | 304 | 0.861 | 0.148 | 0.217 | 13.23 | < 0.001 | 1.17 | 522 |
| Low income | 165 | 0.708 | 0.243 |  | 359 | 0.798 | 0.192 | 0.09 | 4.57 | < 0.001 | 0.43 | 522 |

Cohen’s *d:* 0.2-0.49= small, 0.5-0.79 = moderate, above 0.8 = large

# Table S3b - Known groups validity analysis by gender

|  | **Yes** | | |  | **No** | | | **Mean** |  |  | **Cohen's** |  |
| --- | --- | --- | --- | --- | --- | --- | --- | --- | --- | --- | --- | --- |
| **FEMALE** | **n** | **Mean** | **SD** |  | **n** | **Mean** | **SD** | **difference** | ***t*** | ***p*-value** | ***d*** | **df** |
| **EQ-HWB-S sum-score** |  |  |  |  |  |  |  |  |  |  |  |  |
| Caregiver | 240 | 21.583 | 7.002 |  | 1072 | 19.174 | 7.547 | -2.409 | -4.53 | < 0.001 | -0.32 | 1310 |
| Mental health distress | 331 | 28.356 | 4.916 |  | 981 | 16.665 | 5.718 | -11.691 | -33.28 | < 0.001 | -2.12 | 1310 |
| Chronic health condition | 663 | 21.306 | 7.451 |  | 649 | 17.886 | 7.165 | -3.42 | -8.47 | < 0.001 | -0.47 | 1310 |
| Disability | 177 | 23.492 | 7.718 |  | 1135 | 19.01 | 7.292 | -4.482 | -7.55 | < 0.001 | -0.61 | 1310 |
| Sleep issues | 675 | 23.43 | 6.906 |  | 637 | 15.571 | 5.81 | -7.859 | -22.24 | < 0.001 | -1.23 | 1310 |
| Low income | 416 | 19.589 | 7.674 |  | 896 | 19.626 | 7.431 | 0.037 | 0.08 | 0.934 | 0 | 1310 |
| **EQ-HWB-S index-score** |  |  |  |  |  |  |  |  |  |  |  |  |
| Caregiver | 240 | 0.656 | 0.241 |  | 1072 | 0.736 | 0.231 | 0.08 | 4.77 | < 0.001 | 0.34 | 1310 |
| Mental health distress | 331 | 0.463 | 0.204 |  | 981 | 0.808 | 0.172 | 0.345 | 30.14 | < 0.001 | 1.92 | 1310 |
| Chronic health condition | 663 | 0.654 | 0.246 |  | 649 | 0.789 | 0.201 | 0.135 | 10.86 | < 0.001 | 0.6 | 1310 |
| Disability | 177 | 0.547 | 0.269 |  | 1135 | 0.748 | 0.217 | 0.201 | 11.07 | < 0.001 | 0.89 | 1310 |
| Sleep issues | 675 | 0.61 | 0.236 |  | 637 | 0.839 | 0.166 | 0.229 | 20.14 | < 0.001 | 1.11 | 1310 |
| Low income | 416 | 0.704 | 0.249 |  | 896 | 0.729 | 0.227 | 0.025 | 1.78 | 0.075 | 0.11 | 1310 |
|  |  |  |  |  |  |  |  |  |  |  |  |  |
|  |  |  |  |  |  |  |  |  |  |  |  |  |
|  | **Yes** | | |  | **No** | | | **Mean** |  |  | **Cohen's** |  |
| **MALE** | **n** | **Mean** | **SD** |  | **n** | **Mean** | **SD** | **difference** | ***t*** | ***p*-value** | ***d*** | **df** |
| **EQ-HWB-S sum-score** |  |  |  |  |  |  |  |  |  |  |  |  |
| Caregiver | 238 | 20.176 | 5.947 |  | 982 | 16.702 | 6.865 | -3.474 | -7.18 | < 0.001 | -0.52 | 1218 |
| Mental health distress | 184 | 27.201 | 4.92 |  | 1036 | 15.635 | 5.526 | -11.566 | -26.58 | < 0.001 | -2.13 | 1218 |
| Chronic health condition | 565 | 19.255 | 7.14 |  | 655 | 15.762 | 6.118 | -3.493 | -9.2 | < 0.001 | -0.53 | 1218 |
| Disability | 154 | 20.935 | 7.428 |  | 1066 | 16.866 | 6.591 | -4.069 | -7.04 | < 0.001 | -0.61 | 1218 |
| Sleep issues | 496 | 21.45 | 6.771 |  | 724 | 14.591 | 5.311 | -6.859 | -19.78 | < 0.001 | -1.15 | 1218 |
| Low income | 254 | 17.783 | 7.819 |  | 966 | 17.273 | 6.551 | -0.51 | -1.06 | 0.290 | -0.07 | 1218 |
| **EQ-HWB-S index-score** |  |  |  |  |  |  |  |  |  |  |  |  |
| Caregiver | 238 | 0.71 | 0.185 |  | 982 | 0.797 | 0.21 | 0.087 | 5.89 | < 0.001 | 0.43 | 1218 |
| Mental health distress | 184 | 0.485 | 0.202 |  | 1036 | 0.833 | 0.16 | 0.348 | 25.96 | < 0.001 | 2.08 | 1218 |
| Chronic health condition | 565 | 0.713 | 0.228 |  | 655 | 0.838 | 0.169 | 0.125 | 10.96 | < 0.001 | 0.63 | 1218 |
| Disability | 154 | 0.623 | 0.257 |  | 1066 | 0.803 | 0.19 | 0.18 | 10.49 | < 0.001 | 0.9 | 1218 |
| Sleep issues | 496 | 0.663 | 0.227 |  | 724 | 0.861 | 0.148 | 0.198 | 18.46 | < 0.001 | 1.08 | 1218 |
| Low income | 254 | 0.745 | 0.256 |  | 966 | 0.79 | 0.193 | 0.045 | 3.06 | 0.002 | 0.22 | 1218 |

Cohen’s *d:* 0.2-0.49= small, 0.5-0.79 = moderate, above 0.8 = large

# Table S3c - Known groups validity analysis by age group

|  | **Yes** | | |  | **No** | | | **Mean** |  |  | **Cohen's** |  |
| --- | --- | --- | --- | --- | --- | --- | --- | --- | --- | --- | --- | --- |
| **18-29** | **n** | **Mean** | **SD** |  | **n** | **Mean** | **SD** | **difference** | ***t*** | ***p*-value** | ***d*** | **df** |
| **EQ-HWB-S sum-score** |  |  |  |  |  |  |  |  |  |  |  |  |
| Caregiver | 109 | 23.771 | 5.102 |  | 432 | 21.146 | 7.094 | -2.625 | -3.63 | < 0.001 | -0.39 | 539 |
| Mental health distress | 200 | 27.535 | 4.402 |  | 341 | 18.238 | 5.517 | -9.297 | -20.34 | < 0.001 | -1.81 | 539 |
| Chronic health condition | 212 | 24.151 | 6.325 |  | 329 | 20.079 | 6.654 | -4.072 | -7.08 | < 0.001 | -0.62 | 539 |
| Disability | 36 | 25.556 | 6.059 |  | 505 | 21.398 | 6.79 | -4.158 | -3.57 | < 0.001 | -0.62 | 539 |
| Sleep issues | 293 | 24.922 | 5.589 |  | 248 | 17.839 | 6.117 | -7.083 | -14.06 | < 0.001 | -1.21 | 539 |
| Low income | 110 | 22.8 | 7.094 |  | 431 | 21.387 | 6.724 | -1.413 | -1.94 | 0.052 | -0.21 | 539 |
| **EQ-HWB-S index-score** |  |  |  |  |  |  |  |  |  |  |  |  |
| Caregiver | 109 | 0.602 | 0.168 |  | 432 | 0.694 | 0.223 | 0.092 | 4.03 | < 0.001 | 0.43 | 539 |
| Mental health distress | 200 | 0.496 | 0.176 |  | 341 | 0.781 | 0.162 | 0.285 | 19.15 | < 0.001 | 1.71 | 539 |
| Chronic health condition | 212 | 0.588 | 0.211 |  | 329 | 0.732 | 0.201 | 0.144 | 7.98 | < 0.001 | 0.7 | 539 |
| Disability | 36 | 0.536 | 0.209 |  | 505 | 0.686 | 0.214 | 0.15 | 4.07 | < 0.001 | 0.7 | 539 |
| Sleep issues | 293 | 0.58 | 0.195 |  | 248 | 0.789 | 0.184 | 0.209 | 12.78 | < 0.001 | 1.1 | 539 |
| Low income | 110 | 0.637 | 0.229 |  | 431 | 0.686 | 0.212 | 0.049 | 2.11 | 0.036 | 0.22 | 539 |
|  |  |  |  |  |  |  |  |  |  |  |  |  |
|  |  |  |  |  |  |  |  |  |  |  |  |  |
|  | **Yes** | | |  | **No** | | | **Mean** |  |  | **Cohen's** |  |
| **30-39** | **n** | **Mean** | **SD** |  | **n** | **Mean** | **SD** | **difference** | ***t*** | ***p*-value** | ***d*** | **df** |
| **EQ-HWB-S sum-score** |  |  |  |  |  |  |  |  |  |  |  |  |
| Caregiver | 101 | 21.287 | 6.284 |  | 390 | 19.744 | 7.101 | -1.543 | -1.99 | 0.047 | -0.22 | 489 |
| Mental health distress | 132 | 27.311 | 4.923 |  | 359 | 17.396 | 5.566 | -9.915 | -18.03 | < 0.001 | -1.84 | 489 |
| Chronic health condition | 211 | 22.654 | 6.219 |  | 280 | 18.107 | 6.864 | -4.547 | -7.56 | < 0.001 | -0.69 | 489 |
| Disability | 32 | 24.125 | 7.636 |  | 459 | 19.778 | 6.833 | -4.347 | -3.45 | 0.001 | -0.63 | 489 |
| Sleep issues | 242 | 23.583 | 6.471 |  | 249 | 16.639 | 5.588 | -6.944 | -12.74 | < 0.001 | -1.15 | 489 |
| Low income | 61 | 21.164 | 7.563 |  | 430 | 19.905 | 6.868 | -1.259 | -1.32 | 0.186 | -0.18 | 489 |
| **EQ-HWB-S index-score** |  |  |  |  |  |  |  |  |  |  |  |  |
| Caregiver | 101 | 0.681 | 0.198 |  | 390 | 0.733 | 0.215 | 0.052 | 2.22 | 0.027 | 0.25 | 489 |
| Mental health distress | 132 | 0.497 | 0.188 |  | 359 | 0.805 | 0.153 | 0.308 | 18.56 | < 0.001 | 1.89 | 489 |
| Chronic health condition | 211 | 0.642 | 0.21 |  | 280 | 0.783 | 0.194 | 0.141 | 7.74 | < 0.001 | 0.71 | 489 |
| Disability | 32 | 0.589 | 0.241 |  | 459 | 0.732 | 0.208 | 0.143 | 3.71 | < 0.001 | 0.68 | 489 |
| Sleep issues | 242 | 0.62 | 0.216 |  | 249 | 0.822 | 0.154 | 0.202 | 11.93 | < 0.001 | 1.08 | 489 |
| Low income | 61 | 0.683 | 0.234 |  | 430 | 0.728 | 0.209 | 0.045 | 1.55 | 0.121 | 0.21 | 489 |
|  |  |  |  |  |  |  |  |  |  |  |  |  |
|  |  |  |  |  |  |  |  |  |  |  |  |  |
|  | **Yes** | | |  | **No** | | | **Mean** |  |  | **Cohen's** |  |
| **40-49** | **n** | **Mean** | **SD** |  | **n** | **Mean** | **SD** | **difference** | ***t*** | ***p*-value** | ***d*** | **df** |
| **EQ-HWB-S sum-score** |  |  |  |  |  |  |  |  |  |  |  |  |
| Caregiver | 92 | 21.12 | 6.302 |  | 335 | 18.116 | 7.088 | -3.004 | -3.68 | < 0.001 | -0.43 | 425 |
| Mental health distress | 80 | 28.087 | 4.478 |  | 347 | 16.614 | 5.613 | -11.473 | -17.07 | < 0.001 | -2.12 | 425 |
| Chronic health condition | 176 | 21.824 | 6.769 |  | 251 | 16.618 | 6.394 | -5.206 | -8.08 | < 0.001 | -0.79 | 425 |
| Disability | 58 | 23.948 | 7.299 |  | 369 | 17.949 | 6.635 | -5.999 | -6.31 | < 0.001 | -0.89 | 425 |
| Sleep issues | 193 | 22.845 | 6.329 |  | 234 | 15.397 | 5.668 | -7.448 | -12.82 | < 0.001 | -1.25 | 425 |
| Low income | 66 | 21.561 | 7.722 |  | 361 | 18.252 | 6.782 | -3.309 | -3.56 | < 0.001 | -0.48 | 425 |
| **EQ-HWB-S index-score** |  |  |  |  |  |  |  |  |  |  |  |  |
| Caregiver | 92 | 0.678 | 0.231 |  | 335 | 0.772 | 0.208 | 0.094 | 3.74 | < 0.001 | 0.44 | 425 |
| Mental health distress | 80 | 0.468 | 0.191 |  | 347 | 0.817 | 0.162 | 0.349 | 16.79 | < 0.001 | 2.08 | 425 |
| Chronic health condition | 176 | 0.652 | 0.231 |  | 251 | 0.822 | 0.174 | 0.17 | 8.66 | < 0.001 | 0.85 | 425 |
| Disability | 58 | 0.553 | 0.264 |  | 369 | 0.783 | 0.19 | 0.23 | 8.1 | < 0.001 | 1.14 | 425 |
| Sleep issues | 193 | 0.634 | 0.222 |  | 234 | 0.849 | 0.154 | 0.215 | 11.73 | < 0.001 | 1.14 | 425 |
| Low income | 66 | 0.64 | 0.278 |  | 361 | 0.772 | 0.196 | 0.132 | 4.66 | < 0.001 | 0.62 | 425 |
|  |  |  |  |  |  |  |  |  |  |  |  |  |
|  |  |  |  |  |  |  |  |  |  |  |  |  |
|  | **Yes** | | |  | **No** | | | **Mean** |  |  | **Cohen's** |  |
| **50-59** | **n** | **Mean** | **SD** |  | **n** | **Mean** | **SD** | **difference** | ***t*** | ***p*-value** | ***d*** | **df** |
| **EQ-HWB-S sum-score** |  |  |  |  |  |  |  |  |  |  |  |  |
| Caregiver | 88 | 19.727 | 6.581 |  | 304 | 17.355 | 7.564 | -2.372 | -2.66 | 0.008 | -0.32 | 390 |
| Mental health distress | 57 | 29.702 | 5.467 |  | 335 | 15.878 | 5.607 | -13.824 | -17.27 | < 0.001 | -2.47 | 390 |
| Chronic health condition | 185 | 20.773 | 7.517 |  | 207 | 15.309 | 6.298 | -5.464 | -7.83 | < 0.001 | -0.79 | 390 |
| Disability | 58 | 23.31 | 8.31 |  | 334 | 16.946 | 6.833 | -6.364 | -6.33 | < 0.001 | -0.9 | 390 |
| Sleep issues | 200 | 21.435 | 7.341 |  | 192 | 14.193 | 5.426 | -7.242 | -11.07 | < 0.001 | -1.12 | 390 |
| Low income | 81 | 21.938 | 8.664 |  | 311 | 16.833 | 6.673 | -5.105 | -5.74 | < 0.001 | -0.72 | 390 |
| **EQ-HWB-S index-score** |  |  |  |  |  |  |  |  |  |  |  |  |
| Caregiver | 88 | 0.722 | 0.223 |  | 304 | 0.774 | 0.247 | 0.052 | 1.76 | 0.079 | 0.21 | 390 |
| Mental health distress | 57 | 0.391 | 0.241 |  | 335 | 0.825 | 0.178 | 0.434 | 16.06 | < 0.001 | 2.3 | 390 |
| Chronic health condition | 185 | 0.661 | 0.264 |  | 207 | 0.852 | 0.18 | 0.191 | 8.43 | < 0.001 | 0.85 | 390 |
| Disability | 58 | 0.52 | 0.306 |  | 334 | 0.804 | 0.203 | 0.284 | 9.05 | < 0.001 | 1.29 | 390 |
| Sleep issues | 200 | 0.658 | 0.264 |  | 192 | 0.87 | 0.158 | 0.212 | 9.6 | < 0.001 | 0.97 | 390 |
| Low income | 81 | 0.615 | 0.305 |  | 311 | 0.8 | 0.208 | 0.185 | 6.42 | < 0.001 | 0.8 | 390 |
|  |  |  |  |  |  |  |  |  |  |  |  |  |
|  |  |  |  |  |  |  |  |  |  |  |  |  |
|  | **Yes** | | |  | **No** | | | **Mean** |  |  | **Cohen's** |  |
| **60+** | **n** | **Mean** | **SD** |  | **n** | **Mean** | **SD** | **difference** | ***t*** | ***p*-value** | ***d*** | **df** |
| **EQ-HWB-S sum-score** |  |  |  |  |  |  |  |  |  |  |  |  |
| Caregiver | 89 | 17.854 | 7.043 |  | 602 | 14.963 | 6.402 | -2.891 | -3.92 | < 0.001 | -0.45 | 689 |
| Mental health distress | 52 | 29.115 | 6.233 |  | 639 | 14.214 | 5.16 | -14.901 | -19.69 | < 0.001 | -2.84 | 689 |
| Chronic health condition | 451 | 16.845 | 6.998 |  | 240 | 12.5 | 4.413 | -4.345 | -8.74 | < 0.001 | -0.7 | 689 |
| Disability | 151 | 20.205 | 7.419 |  | 540 | 13.974 | 5.584 | -6.231 | -11.22 | < 0.001 | -1.03 | 689 |
| Sleep issues | 252 | 19.77 | 7.622 |  | 439 | 12.79 | 4.073 | -6.98 | -15.68 | < 0.001 | -1.24 | 689 |
| Low income | 353 | 16.139 | 6.711 |  | 338 | 14.497 | 6.289 | -1.642 | -3.31 | 0.001 | -0.25 | 689 |
| **EQ-HWB-S index-score** |  |  |  |  |  |  |  |  |  |  |  |  |
| Caregiver | 89 | 0.749 | 0.239 |  | 602 | 0.825 | 0.208 | 0.076 | 3.12 | 0.002 | 0.35 | 689 |
| Mental health distress | 52 | 0.392 | 0.263 |  | 639 | 0.849 | 0.168 | 0.457 | 17.93 | < 0.001 | 2.59 | 689 |
| Chronic health condition | 451 | 0.761 | 0.235 |  | 240 | 0.916 | 0.111 | 0.155 | 9.69 | < 0.001 | 0.77 | 689 |
| Disability | 151 | 0.625 | 0.261 |  | 540 | 0.868 | 0.164 | 0.243 | 13.93 | < 0.001 | 1.28 | 689 |
| Sleep issues | 252 | 0.679 | 0.262 |  | 439 | 0.893 | 0.126 | 0.214 | 14.42 | < 0.001 | 1.14 | 689 |
| Low income | 353 | 0.789 | 0.223 |  | 338 | 0.842 | 0.201 | 0.053 | 3.25 | 0.001 | 0.25 | 689 |

Cohen’s *d:* 0.2-0.49= small, 0.5-0.79 = moderate, above 0.8 = large

# Table S4a – Convergent validity analysis by country - Australia

|  | Nervous | Hopeless | Restless or fidgety | Depressed | Everything was an effort | Worthless | K6 total |
| --- | --- | --- | --- | --- | --- | --- | --- |
| Mobility | .338 | .343 | .303 | .400 | .312 | .372 | .408 |
| Activities | .391 | .464 | .407 | .486 | .452 | .494 | .530 |
| Exhaustion | .542 | .555 | .590 | .543 | .626 | .567 | .679 |
| Loneliness | .571 | .631 | .554 | .656 | .582 | .673 | .717 |
| Cognition | .619 | .621 | .663 | .611 | .640 | .635 | .749 |
| Anxiety | .702 | .658 | .685 | .650 | .635 | .664 | .786 |
| Sad/depression | .607 | .689 | .592 | .725 | .632 | .734 | .780 |
| Control | .591 | .661 | .592 | .660 | .624 | .688 | .746 |
| Pain | .206 | .256 | .261 | .238 | .249 | .269 | .294 |
| EQ-HWB-S sum-score | .693 | .735 | .705 | .746 | .721 | .765 | .861 |
| EQ-HWB-S index-score | -.634 | -.686 | -.645 | -.702 | -.666 | -.719 | -.800 |

Cohen’s *d:* 0.2-0.49= small, 0.5-0.79 = moderate, above 0.8 = large

# Table S4b – Convergent validity analysis by country – New Zealand

|  | Nervous | Hopeless | Restless or fidgety | Depressed | Everything was an effort | Worthless | K6 total |
| --- | --- | --- | --- | --- | --- | --- | --- |
| Mobility | .103 | .153 | .163 | .178 | .149 | .115 | .176 |
| Activities | .265 | .276 | .305 | .268 | .350 | .254 | .348 |
| Exhaustion | .509 | .466 | .533 | .430 | .573 | .396 | .589 |
| Loneliness | .553 | .631 | .554 | .614 | .581 | .637 | .706 |
| Cognition | .609 | .585 | .655 | .552 | .610 | .566 | .715 |
| Anxiety | .773 | .625 | .656 | .557 | .580 | .566 | .754 |
| Sad/depression | .600 | .716 | .581 | .733 | .599 | .697 | .770 |
| Control | .546 | .647 | .558 | .594 | .527 | .578 | .687 |
| Pain | .152 | .149 | .232 | .157 | .209 | .160 | .223 |
| EQ-HWB-S sum-score | .670 | .681 | .680 | .647 | .681 | .628 | .804 |
| EQ-HWB-S index-score | -.590 | -.612 | -.610 | -.597 | -.627 | -.574 | -.728 |

Cohen’s *d:* 0.2-0.49= small, 0.5-0.79 = moderate, above 0.8 = large


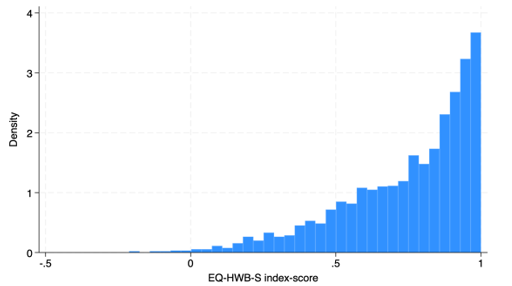


# Figure S1 Distribution of EQ-HWB-S index-scores
